# MyomiRNAs Dysregulation in ALS Rehabilitation

**DOI:** 10.3390/brainsci9010008

**Published:** 2019-01-10

**Authors:** Valentina Pegoraro, Antonio Merico, Corrado Angelini

**Affiliations:** Fondazione Ospedale San Camillo IRCCS, via Alberoni 70, 30126 Venezia, Italy; valentina.pegoraro@ospedalesancamillo.net (V.P.); antonio.merico@ospedalesancamillo.net (A.M.)

**Keywords:** ALS, ALS rehabilitation, myomiRs, circulating miRNAs, muscle, motor neuron

## Abstract

Amyotrophic lateral sclerosis (ALS) is a rare, progressive, neurodegenerative disorder caused by degeneration of upper and lower motor neurons. The disease process leads, because of lower motor neuron involvement, to progressive muscle atrophy, weakness, and fasciculations and for the upper motor neuron involvement leads to spasticity. Muscle atrophy in ALS is caused by a neural dysregulation in the molecular network controlling fast and slow muscle fibers. Denervation and reinnervation processes in skeletal muscle occur in the course of ALS and are modulated by rehabilitation. MicroRNAs (miRNAs) are small, non-coding RNAs that are involved in different biological functions under various pathophysiological conditions. MiRNAs can be secreted by various cell types and they are markedly stable in body fluids. MiR-1, miR-133 a miR-133b, and miR-206 are called “myomiRs” and are considered markers of myogenesis during muscle regeneration and contribute to neuromuscular junction stabilization or sprouting. We observed a positive effect of a standard aerobic exercise rehabilitative protocol conducted for six weeks in 18 ALS patients during hospitalization in our center. This is a preliminary study, in which we correlated clinical scales with molecular data on myomiRs. After six weeks of moderate aerobic exercise, we found lower levels in serum of myomiRNAs. Our data suggest that circulating miRNAs changed during skeletal muscle recovery in response to physical rehabilitation in ALS. However, no firm conclusions can be made on the ALS-specific effect of exercise on miRNA levels.

## 1. Introduction

Amyotrophic lateral sclerosis (ALS) is a rare, progressive, neurodegenerative disease that involves both lower motor neurons in the spinal cord or brainstem and upper motor neurons. Loss of motor neurons leads to muscle atrophy and weakness, fasciculations, and spasticity [1]. Approximately two-thirds of patients with ALS have the classical ‘spinal form’ of the disease [2], with onset in lower limbs and symptoms associated with muscle atrophy. Patients with a bulbar onset of ALS initially exhibit dysarthria and dysphagia for solids and liquids [2]. In ALS, death occurs within 3–5 years but there is great variability in the duration of the disease, since some patients die a few months after the onset and others survive for more than twenty years [1].

The causes of sporadic ALS probably occurs as a result of complex interactions between environmental mechanisms and the activation of ALS pathogenetic mechanisms, such as oxidative stress, defects in RNA processes, mitochondrial dysfunction, protein aggregates, excitotoxicity, problems in axonal transport, and inflammation [3,4,5,6]. Pathogenic processes leading to the disease involve both motor-neurons and non-neuronal cells, including astrocytes, microglia, T-cells, and skeletal muscle. Muscle atrophy in ALS is caused by a dysregulation in the “molecular network” of autophagy, mitochondrial biogenesis, the proliferation of satellite cells, and muscle regeneration processes [7,8]. Therefore, structural and metabolic changes in skeletal muscle can aggravate the course of the disease. Recent studies suggest that skeletal muscle contributes to a retrograde signaling cascade that impairs motor neurons [9,10,11].

In a clinical trial, we found that regular, rehabilitative exercise in ALS patients helped to reduce pain and fatigue of skeletal muscle origin [12]. If a patient is inactive, the loss of training and disuse leads to muscular atrophy, which adds to the weakness and muscular atrophy caused by denervation and degeneration of motor neurons in ALS.

MicroRNAs negatively regulate gene expression at the post-transcriptional level [13] by pairing with specific messenger RNAs (mRNAs), leading to degradation or preventing translation into the corresponding protein product. The up-regulation of a specific miRNA determines a decrease in the expression of the corresponding protein product. Bioinformatic predictions indicate that mammalian miRNAs could regulate more than 30% of all proteins [14].

MiRNAs are involved in a wide range of physiological and pathological processes and their dysregulation is observed in several human diseases. Dysregulation of miRNAs has been documented in ALS [15,16,17] as well as in Alzheimer’s, Huntington’s, and Parkinson’s diseases [18,19].

Different studies underlined the importance of miRNAs in the control of skeletal muscle development and function. Muscle-specific miRNAs miR-1, miR-206, miR-133a, and miR-133b, called together “Canonical myomiRs”, have been well identified and characterized. They modulate fundamental aspects of muscle biology, such as myogenesis (including myoblast or satellite cell proliferation and differentiation), apoptosis, and have a role as muscle mass modulators [20,21].

MyomiRs are predominantly expressed in cardiac and skeletal muscle [22,23]. MiR-1 and miR-206 are involved in the regulation of muscle transcription factors, such as MyoD and myogenin. MiR-1 and miR-206 promote myogenesis, respectively, by targeting histone deacetylase 4 (HDAC4) and by downregulating paired box 7 (Pax7). In contrast, the miR-133 family inhibits differentiation and contributes to the proliferation of myoblasts by repressing Serum Response Factor (SRF) [24]. MiR-133b is dysregulated in ALS muscle and ALS spinal cord [25]. In addition, miR-206 and miR-133b have a role in the development and maturation of neuromuscular synapses [26].

MiRNAs are promising potential diagnostic biomarkers and they could be utilized to monitor the progression of the disease and to evaluate responses to rehabilitative treatments [27].

In this preliminary study, we correlate observations on the effects of physical rehabilitation in ALS patients. No firm conclusions can be made on the ALS-specific effect of exercise on miRNA levels due to lack of a healthy control group, due to regulatory limitations.

## 2. Materials and Methods

### 2.1. Patient Selection

A cohort of 18 ALS patients was included in this study and diagnosed according to the revised EI Escorial criteria [28]. Inclusion criteria included a sporadic ALS form, and mild to moderate disability. We excluded ALS patients with cardiac involvement, history of other neurological and metabolic disorders, and severe neuropsychiatric illness that caused patients to be unable to understand and perform instructions. The level of physical disability was assessed using the Amyotrophic Lateral Sclerosis Functional Rating Scale-Revised (ALSFRS-R).

The study was performed in accordance with the ethical standards of the Declaration of Helsinki. The investigation and use of patients’ data for research purposes were approved by the San Camillo Foundation research ethical committee in January 2016, in accordance with the Declaration of the World Medical Association.

Serum samples were obtained from peripheral blood of 18 ALS patients after written informed consent. In ALS patients, serum was collected at the beginning of the patient’s admission, hereafter called Time Zero (T0, baseline), and the second time after a period of physical rehabilitation, named Time One (T1). Biological samples were stored frozen at −80 °C in Biobank of Rare Diseases and Neuro-Rehabilitation (BBMRNR) at Foundation Hospital San Camillo IRCCS until use.

### 2.2. Patient Rehabilitation Protocol

The rehabilitation programme consisted of an individualized progressive training of muscular strengthening and aerobic endurance exercises to avoid muscle damage, performed daily for 6 weeks during hospitalization. In relation to their level of disability, the ALS patients performed a cycle ergometer, arm-leg ergometry or treadmill, or standard rehabilitation consisting of a one-hour session of stretching, active mobilization, and general reinforcement, as previously described [12]. In addition, all ALS patients underwent speech, occupational, and psychological therapy.

A series of clinical scales were used to assess patients before and after physical rehabilitation; functional autonomy was evaluated by the Functional Independence Measure (FIM). The Fatigue Severity Scale (FSS) was administered to measure the degree of fatigue and its effect on the patient’s activities. The Barthel Index was used to measure performance improvement in daily life activities.

### 2.3. Sera Collection

We performed venous withdrawals in fasting ALS patients. We collected blood in a Vacutainer tube (BD Vacutainer® SST™ II Advance, Becton Dickinson, Wokingham, UK) with a gel separation for serum. After collection of the whole blood, we allowed the sample to coagulate at room temperature for at least 30 min and was then centrifuged at 1500× *g* for 15 min at room temperature. The resulting supernatant is designated as serum. Following centrifugation, the liquid component (serum) was transferred into a clean polypropylene tube, the serum was divided into 200 µL aliquots, and immediately stored at −80 °C in BBMRNR Biobank until used.

### 2.4. RNA Extraction and MiRNA Quantification

MiRNAs were isolated from 400 µL of serum using the miRNeasy Mini Kit (Qiagen, Hilden, Germany) following the instructions of the manufacturer. QIAzol Lysis reagent was added to 400 µL of a serum sample to denaturated protein complexes and RNases. After 15 min incubation at room temperature, one volume of chloroform and 10 µL of 5nM of miR-39-3p of *C. elegans* were added. The lysate was separated into aqueous and organic phases by centrifugation. The aqueous phase was transferred to a new collection tube and ethanol was added. The solution obtained was transferred to the RNeasy MinElute spin column, where the total RNA binds to the membrane while phenol and other contaminants are efficiently washed away. RNA was then eluated in RNase-free water.

Quantification of miRNA levels were determined using a quantitative real-time polymerase chain reaction (qRT-PCR) with the CFX96™ Real-Time PCR detection System (Biorad, Hercules, CA, USA) and TaqMan MicroRNA Assay: hsa-miR-1 (assay ID 002222), hsa-miR-133a (assay ID 002246), hsa-miR-133b (assay ID 002247), hsa-miR-206 (assay ID 000510), cel-miR-39-3p (assay ID 000200) (Applied Biosystems, Carlsbad, CA, USA). One microliter of total RNA was reverse transcribed with a TaqMan MicroRNA Reverse Transcription Kit (Applied Biosystems, Carlsbad, CA, USA) in a total volume of 15 μL. The resulting cDNA was amplified by Real-Time PCR in a total volume of 20 μL, according to the manufacturer’s protocol. Amplification was carried out as follows: 50 °C for 2 min, 95 °C for 10 min, 40 cycles of 95 °C for 15 seconds, and 60 °C for 1 min. All reactions were carried out in triplicate. MiRNA levels were calculated using the ΔΔCT method. The normalization of miRNA levels was performed with miR-39-3p of *C. elegans*, which as previously described [16,29] was added as a spike-in control to measure the efficiency of RNA extraction, reverse transcription, and PCR amplification. Baseline data, or Time Zero (T0) before rehabilitation treatment, were set as a control to calculate fold change using the 2-ΔΔCt method of Time One (T1); qPCR data are presented in the box-plot as a relative expression from the pre-training level where ‹1 equals a reduction in miRNAs levels.

### 2.5. Statistical Analysis

We used the Wilcoxon-Mann-Whitney test for paired data for small samples to verify the validity of data obtained. The level of significance was set at *p* < 0.05. Data were analyzed using the R-studio program (R version 3.4.4. 2018 for Windows, R Foundation for Statistical Computing, Vienna, Austria).

## 3. Results

### 3.1. Patient Cohort and Selection

Eighteen patients were selected from a cohort of twenty-one ALS patients admitted to Foundation Hospital San Camillo IRCCS (Venice, Italy) and were evaluated in the course of 6 weeks of physical rehabilitation. Eighteen patients who met the inclusion criteria, described in the materials and methods, were enrolled in the study. The clinical features of ALS patients are reported in Table 1. The ALS patients (eleven males and seven females), had an average age of 61.1 years with a mild or moderate disability score, with an ALSFRS-R mean average of 34.6 ± 4.9.

We collected clinical scales and peripheral blood from selected ALS patients before (T0) and after (T1) a period of six weeks of training.

In Table 2 we report sex, age, and the values of clinical outcomes, according to scale measurements recorded at T0 and at T1 for every ALS patient. After physical training, the ALS patients showed an improvement of muscle strength, an improvement of physical conditions, and independence documented by a significant change (*p*-value ≤ 0.05) in ALSFRS-R, Barthel, and FIM scores and a decreased sense of fatigue observed by FSS in T1 (*p*-value ≤ 0.05), underlining the positive effect of rehabilitation.

### 3.2. Circulating MyomiRNAs

**Figure 1:**
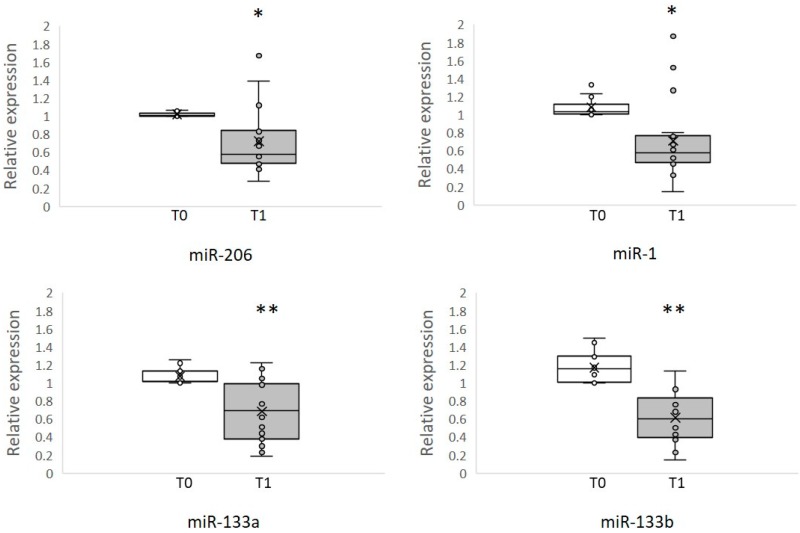
Differential levels of circulating myomiRs in eighteen ALS patients following 6 weeks of physical rehabilitation. The graphs show the levels of miR-206, miR-1, miR-133a, and miR-133b in the serum of ALS patients during rehabilitation. We observed significantly lower levels of miR-206 (* *p*-value < 0.05), miR-1 (* *p*-value < 0.05), miR-133a (** *p*-value < 0.001), and miR-133b (** *p*-value < 0.001) after rehabilitation treatment (T1). The qPCR data are presented as a relative expression from the pre-training level (T0), where <1 equals a reduction in miRNAs levels. A decreasing trend of miRNAs was observed after rehabilitation training, suggesting a modulation of myomiRs with training. In the box plot, the line that divides the box into two equal parts is the median. Half the scores are greater than or equal to this value and half are less. Individual levels of each myomiRs at T0 and T1 are shown with a single point, while the average of the data is represented with an X. Some values overlap and do not appear in the box-plot.

We measured circulating muscle-specific miRNAs by qRT-PCR in the serum of eighteen ALS patients before (T0) and after (T1) 6 weeks of physical rehabilitation. To analyze the T1 of each patient, the myomiRNA level was calculated as a relative expression from patient baseline. We found significantly lower levels of miR-1, miR-206, miR-133a, and miR-133b after physical training, as shown in Figure 1. We propose that the decrease in levels of myomiRs that we found after training was due to stabilization of skeletal muscle and neuromuscular junction (NMJ) of ALS patients.

A general decrease of miRNA was observed after training; for miR-206 we observed lower levels in 13 patients. MiR-1 appeared to decrease in 15 patients. Three patients (pt 13-14-17) for miR-1 and one (pt 13) for miR-206 were different and were outside of the upper quartile at T1. In these patients we observed an increase in the level of these two miRNA however two of them were using a deambulator. In our ALS patients, we found a similar trend in miR-1 and miR-206 and in miR-133a and miR-133b after training.

## 4. Discussion

Current medical treatment for ALS is limited to supportive care, and few drugs have been approved for ALS treatment that modestly delay disease progression (i.e., riluzole, endavarone), while other symptomatic treatments, such as dextromethorphan and quinidine, are directed to reduce bulbar or similar symptoms [30]. In recent years, the role of skeletal muscle involvement in ALS has been discussed and muscle-specific miRNAs are emerging as biomarkers [31]. In this pilot study, we examined the effects of physical rehabilitation in patients with ALS. We collected as outcome clinical scales and studied sera miRNA before and after training. The effects of physical rehabilitation in patients with ALS are under investigation, but moderate and regular exercise is supported in the treatment of numerous muscle conditions [32,33]. Patients with ALS are often advised to avoid exercise in order to preserve muscle strength and to minimize the effects of possible muscle overload. This precaution is based on epidemiological studies showing a high incidence of ALS in people who did an intense physical sporting activity, such as soccer players. This activity does not appear equivalent to exercise in rehabilitation [34,35]. Animal models and human studies reinforce the possible benefit of an exercise program, suggesting that moderated endurance exercise can delay disease progression and increase survival. While in ALS mouse models, it was found that physical activity did not affect survival [36], moderate and regular physical activity in ALS people is associated with a temporary positive effect on the disease symptoms [37] and improved functional disability scores [38]. In another study, conducted in a few ALS patients with respiratory failure, exercise resulted in a clinical deterioration [39]. Current studies support the effectiveness of exercise in patients; two recent clinical trials in ALS patients [12,40] demonstrated safety and tolerability of resistance and endurance training. Lunetta and collaborators [41] monitored an exercise program and observed reduced motor deterioration. In healthy people, regular physical activity provides benefits, preventing obesity and heart disease as well as reducing anxiety, pain [33], and fatigue.

In accordance with recent reports, we observed a positive effect of physical training, since ALS patients had an improvement of muscle strength, physical conditions, and independence, documented by a significant change in ALSFRS-R, Barthel, and FIM scores. We also investigated the impact of rehabilitation in terms of fatigue, a common symptom in ALS patients [42] that negatively affects their quality of life. In our patients, there was a significant change in FSS index.

Mouse models of ALS [26,43,44] and spinal muscular atrophy [45] have shown an up-regulation of miR-206, which correlates with the onset of the disease. Muscle atrophy occurs for disorganization of the NMJ, which might slow down because of muscle reinnervation [26].

We previously found an increase of myomiRs in ALS patients compared to controls at rest, both in serum and in muscle biopsies [15,16]. Here we analyzed the levels of muscle-specific microRNAs in the serum of ALS patients after a period of six weeks of moderate physical rehabilitation and we observed significantly lower levels of circulating miR-1, miR-133a, miR-133b, and miR-206 after training. The similar trends observed for miR-1 and miR-206 and for miR-133a and miR-133b in ALS rehabilitation is probably related to their common targets and function in muscle. There was only a lack of decrease in myomiRNAs levels in advanced cases (Figure 1). These few patients used a deambulator and had more atrophic muscles. The possible explanation for their discrepant increased level of myomiRNA is that, while muscle mass increased in most ALS patients, it did not increase in those with fatigue. In an ALS genetic case (ALS-4; pt 14 Table 2), possibly other mechanisms were present.

Several studies underline the function of miR-1, miR-133, and miR-206 in the regulation of muscle mass. MiR-206 was found increased in several muscle atrophy conditions, such as sarcopenia, during space flight and hibernation [16,22,24,46]. The hypertrophy of cultured myotubes induced by inhibition of miR-206 was mediated through de-repression of HDAC4 and it’s suggested that a reduction of miR-206 in myotubes could promote myotube hypertrophy by myoblast fusion. The levels of miR-1 and miR-133 were reduced, respectively, during skeletal muscle hypertrophy and increased during muscle differentiation [47].

In our study there are limitations, since our patient cohort is not large but all 18 of our patients successfully completed 6 weeks of rehabilitation. We also analyzed a homogeneous sample of ALS patients with a moderate grade of disability, as documented by the ALSFRS-R scale. Physical rehabilitation was conducted as inpatients and it was not possible to study healthy people with a similar inpatient protocol. After acute exercise, healthy untrained subjects showed an increased myomiRNAs level similar to a stress reaction, while after long-term exercise training a decrease in miR-1, miR-133a, miR-133b, and miR-206 was found [48,49].

No firm conclusions can be made on the ALS-specific effects of exercise on miRNAs levels. More extensive and better-controlled studies are therefore required in the future to further elucidate benefits of exercise on miRNAs in ALS patients.

## 5. Conclusions

We observed a positive effect of moderate rehabilitative training in our series of ALS patients documented by a significative change in ALSFRS-R and FIM scores and a significant reduction of patient subjective fatigue with a decrement in the FSS scores after exercise. This represents a preliminary study where we observed a decrease in miR-1, miR-133a, miR-133b, and miR-206 after ALS rehabilitation compared to baseline. The observed dysregulation of myomiRNAs might correlate with the clinical response to rehabilitative treatment.

## Figures and Tables

**Table 1:** Clinical characteristics of the ALS patients at baseline.

ALS Features	Mean ± SD
Age (years)	61.1 ± 12.8
Sex M/F	11/7
Disease duration (years)	4.3 ± 3
ALSFRS-R	34.6 ± 4.9

ALS: Amyotrophic lateral sclerosis; M: Male; F: Female; ALSFRS-R: Amyotrophic Lateral Sclerosis Functional Rating Scale-Revised.

**Table 2:** Clinical features and disability scales of ALS patients before (T0) and after (T1) rehabilitation treatment.

Patient	Age	Onset	Sex	ALSFRS-R T0	ALSFRS-R T1	FSS T0	FSS T1	FIM T0	FIM T1	Barthel T0	Barthel T1
Pt 01	74	N.A.	M	29	29	5.40	3.90	78	80	45	60
Pt 02	51	39	M	40	41	5.50	3.90	77	82	50	55
Pt 03	57	53	F	38	40	5.60	4.20	86	90	55	70
Pt 04	79	72	M	29	29	5.30	5.20	85	87	30	40
Pt 05	50	45	M	40	41	5.50	3.90	77	82	50	55
Pt 06	61	58	M	31	31	5.50	5.50	37	36	N.A.	N.A.
Pt 07	45	40	F	28	28	5.50	5.50	88	91	70	80
Pt 08	33	30	F	32	32	5.20	4.80	65	69	35	40
Pt 09	76	70	F	34	34	5.50	4.90	N.A.	65	20	35
Pt 10	44	43	M	32	32	5.40	5.00	83	87	60	65
Pt 11	71	69	M	36	36	5.50	5.10	N.A.	69	20	35
Pt 12	71	57	M	32	33	5.40	3.90	78	84	45	55
Pt 13	61	57	F	33	34	5.10	4.00	60	70	35	45
Pt 14 *	71	66	M	28	28	5.60	4.40	65	N.A.	40	45
Pt 15	65	63	F	39	40	4.50	3.20	83	85	50	55
Pt 16	65	65	F	42	43	5.40	4.20	73	79	45	50
Pt 17	72	62	M	36	36	5.60	4.00	81	89	60	90
Pt 18	54	51	M	43	44	4.80	3.20	80	88	60	70

ALSFRS-R: Amyotrophic Lateral Sclerosis Functional Rating Scale-Revised; FSS: Fatigue Severity Scale; FIM: Functional Independence Measure; * senataxin-mutation (ALS-4); N.A.: not available.
